# National Stop Transmission of Polio Program support for polio supplemental immunization activities in Nigeria 2012-2016: deployment of management support team

**DOI:** 10.11604/pamj.supp.2022.40.1.32562

**Published:** 2022-02-28

**Authors:** Aboyowa Arayuwa Edukugho, Ndadilnasiya Endie Waziri, Omotayo Bolu, Saheed Oluwatoyin Gidado, Lilian Akudo Okeke, Belinda Vernyuy Uba, Jibrin Manu Idris, Charles Akataobi Michael, Joel Oluwasegun Adegoke, Philip Bammeke, Usman Saidu Adamu, Patrick Mboya Nguku, Oladayo Biya, Chima John Ohuanbunwo, John Vertefeuille, Eunice Damisa, Eric Wiesen

**Affiliations:** 1 African Field Epidemiology Network, Abuja, Nigeria,; 2 US Centre for Disease Control and Prevention, Abuja, Nigeria,; 3 National Primary Health Care Development Agency, Abuja, Nigeria,; 4 Center for Disease Control and Prevention, Global Immunization Division, Atlanta

**Keywords:** MST, supervision, polio, NSTOP, NEOC

## Abstract

**Introduction:**

to support polio eradication activities in Nigeria, in 2012 the National Polio Emergency Operation Center (NEOC) created the Management Support Teams (MST) to address gaps in the quality of supervision of polio vaccination teams. The National Stop Transmission of Polio (NSTOP) Program supported the polio eradication activities by deploying trained supervisors as part of the MST for polio and non-polio immunization campaigns.

**Methods:**

trained MST members were deployed approximately 4 days before the start of the campaign to participate in pre-implementation activities and supervise vaccination teams during campaigns. Terms of reference (TOR) developed by NEOC was provided to MST members to guide their activities. Qualified MSTs that met pre-determined criteria were selected and deployed to the field to support pre, intra and post campaigns activities.

**Results:**

a pool of over 400 MST personnel have been identified, trained, and repeatedly deployed from 2012 till 2016. The number of deployed MST personnel rose from 40 per campaign in October 2012 to 342 in May 2016. Of these, 270 (79%) MST personnel were deployed to 11 polio high-risk states of northern Nigeria, where campaigns are conducted between eight and ten times yearly as planned by NEOC. For measles campaigns, about 300 (75%) MST personnel were deployed for the one-off northern and southern campaigns in 2016. The results of clustered Lot Quality Assurance Sampling (LQAS) post-campaign vaccination coverage surveys, a measure of campaign quality, of which introduction into the polio program coincided with deployment of MSTs, showed improvement over time, from 10% (very poor quality) in February 2012 to about 90% (good quality) in December 2016.

**Conclusion:**

the deployment of MST personnel increased the number of trained supervisors in the field, frequency of supervisory visits and had a positive impact on the quality of polio campaigns.

## Introduction

The World Health Assembly launched the Global Polio Eradication Initiative (GPEI) in 1988 and declared polio eradication a programmatic emergency for global public health in 2012 [1]. After years of wild poliovirus (WPV) transmission in Nigeria, the last identified WPV case occurred in September 2016. Supplementary immunization activities (SIAs) are a type of rapid intervention to raise the immunity level of a target population in a given geography and age group within a shorter period than possible through routine immunization (RI) services directed to children under 1 years of age [2]. This strategy has been employed successfully in stopping poliovirus transmission in other parts of the world where routine immunization coverage is low, and is a frequently used tool in Nigeria [3]. In general, two national and two subnational SIAs are conducted annually in Nigeria based on recommendations from the Expert Review Committee (ERC) on polio and routine immunization. One of many obstacles to successful vaccination operations is the lack of human resources to provide quality supervision to vaccination teams [4]. Indeed, one of the lessons learned from the success of global smallpox eradication is the value of massive deployment of trained supervisors to oversee the planning and implementation of vaccination and surveillance activities [5].

In a review of the polio eradication effort by the National Polio Emergency Operation Center (NEOC), the dearth of senior supervisors to provide technical support during immunization campaigns was identified as one of the barriers to attaining high SIA quality regarding vaccination coverage of children under five years of age. To address this gap, NEOC recommended a surge in human capacity to oversee immunization campaign operations [6]. This recommendation gave rise to the concept of a Management Support Team (MST) by NEOC in 2012. Led by the government through the National Primary Healthcare Development Agency (NPHCDA), the Nigeria Polio Eradication Initiative (PEI) was comprised of representatives from all stakeholders: World Health Organization (WHO), United States Centers for Disease Control and Prevention (CDC), National Stop Transmission of Polio (NSTOP), United Nations Children´s Fund (UNICEF), Rotary International and the Core Group Partners Project (CGPP). NPHCDA focused on overall management, WHO on microplan development and validation and the implementation of immunization activities, CDC and NSTOP on reaching underserved communities in those immunization activities, UNICEF on communication and community mobilization, Rotary on advocacy and CGPP on community mobilization. To support this decision on MSTs, NSTOP began training personnel to be deployed during immunization campaigns to provide supervision and technical support. Specifically, these highly trained personnel would ensure that vaccination teams reached all targeted children under 5 years of age aiming 1) no households or settlements were missed from microplanning; 2) access to hard-to-reach, underserved or insecure settlements was increased; 3) non-compliance and other persistent issues were quickly resolved; and 4) community leaders were engaged at all levels.

The NSTOP program was established in July 2012 to provide management and technical support to polio eradication and routine immunization programs in Nigeria, in collaboration with NPHCDA, NEOC and other Global PEI partners, including funding and technical assistance in training by the U.S. CDC. The NSTOP goal is to contribute to the interruption of wild poliovirus transmission and the maintenance of polio eradication. This paper describes how NSTOP deployed a system of senior supervisors as part of MSTs to improve the quality of SIAs. This is an observational assessment of MST deployment during October 2012-May 2016.

## Methods

The first step was to identify qualified personnel to be engaged. Selection was based on being graduates or residents of Nigeria Field Epidemiology and Laboratory Training Program (NFELTP), staff of Ministry of Health or Agriculture, staff of two NFELTP collaborating universities (Ahmadu Bello University Zaria and University of Ibadan) or staffers from other GPEI implementing agencies (Table 1). These candidates included medical doctors, epidemiologists, veterinary doctors, laboratory scientists and nurses. They were trained for two weeks using a curriculum adopted from the CDC international Stop Transmission of Polio (STOP) Program but adapted by NSTOP to reflect the Nigerian PEI context. The curriculum addressed polio epidemiology and GPEI strategies in Nigeria. Topics covered included: planning, conducting, and monitoring polio campaigns, developing effective polio SIA and routine immunization micro-plans, understanding the SOPs for outreach to underserved populations, acute flaccid paralysis (AFP) surveillance, case investigation and outbreak response. Other topics included understanding and supporting the national polio eradication emergency plan (NPEEP), implementing the national accountability framework, strengthening routine immunization systems, conducting non-polio SIAs and the epidemiology and prevention of other vaccine-preventable diseases.

**Table 1:** NSTOP training participants by category and year of training, Nigeria, 2012-2016

Year deployed	Category of trained participants								
	NFELTP Residents	NFELTP Graduates	University staff	FMOH/ NPHCDA	FMARD	State staff	Partner agencies	AFENET Staff	Total
2012	38	11	5	4	2	12	0	0	72
2013	22	9	3	3	2	13	4	0	56
2014	53	9	2	8	2	27	6	4	111
2015	49	4	0	4	2	4	0	9	72
2016	52	3	0	2	2	20	8	8	95
Total	214	36	10	21	10	76	18	21	406

Legend: NFELTP - Nigeria Field Epidemiology and Laboratory Training Program, FMOH - Federal Ministry of Health, FMARD - Federal Ministry of Agriculture, NPHCDA - National Primary Healthcare Development Agency, AFENET - African Field Epidemiology Network

**Figure 1:**
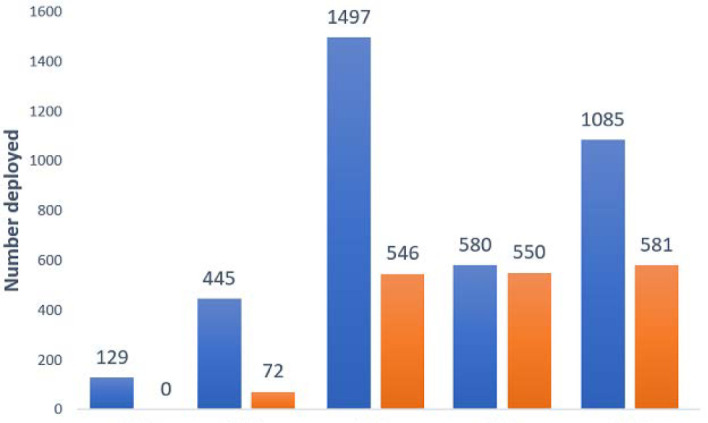
showing the number of deployments per year

Recruited MSTs were trained and deployed during October 2012 and May 2016. Based on the NEOC annual national polio and non-polio SIA campaign calendar, NSTOP deployed these MSTs to support operations. In addition to core NSTOP MSTs, beginning in May 2013, NSTOP provided training and financial support for deploying Nigerian Government MSTs. For NSTOP-deployed MSTs, training, deployment, and funding were done solely by NSTOP whereas for the NSTOP-supported government MSTs, training and deployment was done by government staff while NSTOP provided funding for MST field activities (Figure 1).

Terms of reference (ToRs) were developed to guide the activities of MSTs in the field. Among the activities described in the ToRs were attending state level briefings and orientations with other senior supervisors, developing a work-plan for supervision throughout the duration of the campaign in collaboration with the LGA team, supervising and filling the checklists with at least three vaccination teams daily, and improving the quality of the ward, LGA and state levels evening debriefing by communicating findings and best practices during such meetings. During pre-implementation and implementation activities, MSTs were supervised by NSTOP state team leads. At the end of the campaign, they debriefed the state and provided a written report as part of their accountability. All deployed MSTs received remunerations that covered lodging, transportation, meals, incidentals, and other expenses including phone calls for the period of the field exercise.

**Figure 2:**
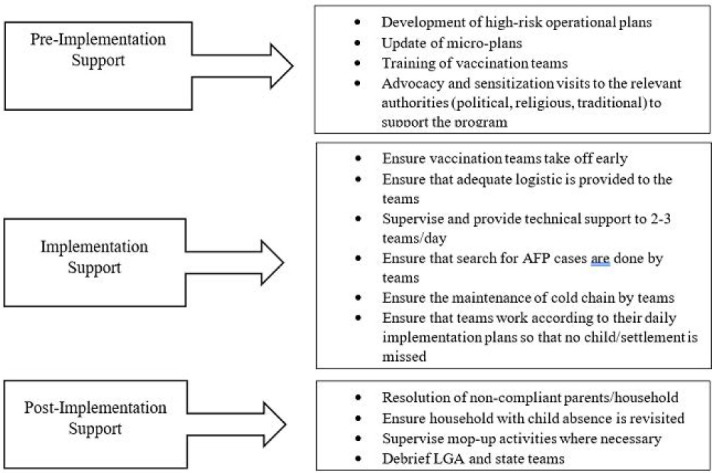
schematic diagram showing MST support during campaign

The scope of the campaign determined the number of MSTs needed for deployment, and the appropriate numbers were selected from a pool of available personnel. Deployment took place 3-4 days before the start of the campaign so MSTs could participate in pre-implementation activities. These activities included; working with state and local government immunization teams to develop high risk operational plans, update micro-plans, participate in trainings of vaccination teams and conducting advocacy and sensitization visits to the relevant authorities (political, religious, traditional) to support the program. The MSTs were in the field throughout implementation to supervise, monitor and provide technical support to vaccination teams. MSTs remained post-implementation to participate in some activities that included resolution of non-compliance by parents, households or groups of households and closing the gaps in follow-up of child absence during SIAs. Figure 2 shows a schematic diagram of MST support for polio SIAs.

The quality of the campaigns implemented were assessed by vaccination coverage surveys using the clustered Lot Quality Assurance Sampling (LQAS) survey method. This method involves counting the number of children out of 60 sampled who had not been vaccinated during the SIA, assessed by a missing finger-mark of vaccination at the time of survey[7]. The official WHO protocol and previous Nigeria results included multiple thresholds, so there were four levels of interpretation of the results. LQAS survey results are interpreted as follows: (a) a 90% threshold is reached whereby 3 or fewer children out of 60 sampled were not finger-marked implies a good quality SIA; (b) a 80% threshold whereby 4 - 8 children were not finger-marked implies a moderate quality SIA; (c) a 60% threshold whereby 9 - 19 children were not finger-marked during the survey implies a poor quality SIAs; and (d) below a threshold of 60% whereby 20 or more children were not finger-marked implies a very poor quality SIA. However, in Nigeria, any result below 90% is considered a failed LQAS survey and a mop-up campaign must be conducted in the settlement within that LGA where the missed children were seen.

The protocol for this assessment was classified as non-research by NPHCDA, the government agency responsible for immunization in Nigeria and was covered by the overall project approval granted to NSTOP.

## Results

**Figure 3:**
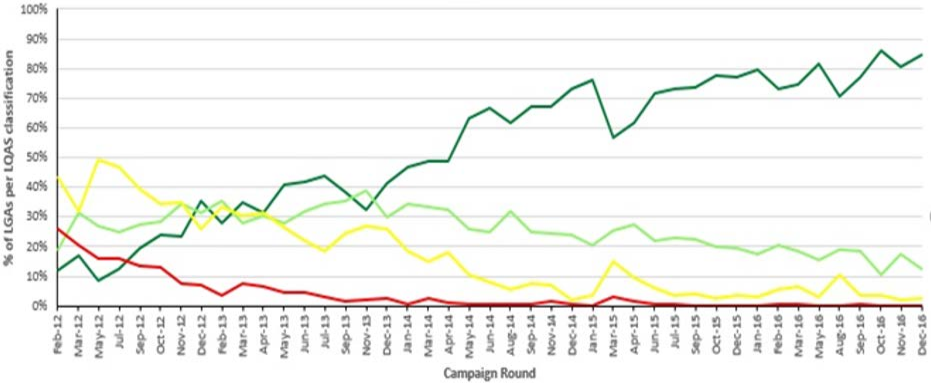
LQAS trend of polio campaigns in Nigeria: 2012-2016

A total of 406 public health professionals were identified and trained as MST workforce between October 2012 and May 2016. Of these, 53% were NFELTP residents. One hundred and eleven (27%) additional individuals were trained in the year 2014, representing the year with the highest number of trained MST personnel. Table 1 shows the category of participants trained during the period under review. The number of NSTOP MST personnel deployed for each campaign round increased from 40 in October 2012 to 342 in May 2016. There was also a steady increase in the number of NSTOP-supported government MST personnel from 2013 to 2016. Figure 2 shows the number of MST personnel deployed by year for OPV SIAs by NSTOP and those deployed by the government but supported by NSTOP. The cumulative number of persons deployed in a particular year was dependent on the number of SIAs held in that year. A review of the LQAS trend within the period of 2012 - 2016, as shown in Figure 3, indicates improvement in polio campaign quality. Higher proportion of passing LQAS (above 90%) means a smaller number of children were missed during the campaign. Only 10% of all LGAs surveyed in February 2012 had a good quality campaign whereas as of December 2016 almost 90% of the LGAs surveyed had a good quality campaign.

## Discussion

The start and acceleration in deployment of MST personnel that improved the supervision of polio SIAs since 2016 coincided with improvement in quality of SIAs as routinely assessed by LQAS survey immediately following every campaign. The increase in the quality of campaigns as shown by the LQAS survey trend (2012 - 2016) correlates chronologically with increase in number of MST personnel deployed to provide supportive supervision for polio campaigns. Although passing the LQAS survey is measured only through inclusion of a mixture of the same and other LGAs over time, it is indicative that fewer children were missed, and more were immunized during this period of targeted MST deployment, thereby reducing the incidence of poliovirus transmission.

The MST model of supportive supervision by senior officers at the national level of frontline field officers involved in SIAs to carry out their duties more effectively has not been documented in a peer-reviewed journal in our literature search. However, supportive supervision has been shown to improve health program performance in other settings. A detailed crossover study of clinical care in hospital settings correlated benefits in the patient care performance of designated nurses when supportive supervision was implemented [8]. A supportive supervision intervention was inferred to increase in assessed essential (routine immunization) coverage in India when appropriate personnel trained for supportive supervision of immunization in the context of implementing WHO´s Reach Every District (RED) strategy [9]. The CDC STOP program has been involved in similar activities through the deployment of high quality public health volunteers to supervise immunization and surveillance activities in countries at high risk of poliovirus transmission [5].

The increase in the quality of SIAs seen during this period as measured by clustered LQAS surveys followed the identification and training of qualified persons to carryout supportive supervision of vaccination teams in the field. Previous attempts in Nigeria to increase supervision have been conducted in past without any significant improvement in coverage [10]. This failure to improve quality via supervision could be possibly associated with inadequate numbers of supervisors deployed to oversee team performance and the possibility of supervisors not properly trained to carry out this supervisory role [10]. The high requirements for health professionals during the selection process to serve as MST personnel (supervisors) in NSTOP MST pool is likely to have ensured higher quality of supervision in the field and strict adherence to and application of SIA guidelines and procedures.

### Limitation

This report has a substantial limitation. This is an observational before and after comparison without a control group. Several improvements and intervention efforts were implemented over the period of observation spearheaded by the Nigerian government via the NEOC with support of GPEI partners in Nigeria. These each could have also substantially contributed to the improvement in quality of polio campaigns over time. Hence, attributing outcome entirely to any one factor would not be appropriate. No other assessments of the quality of the supervision were conducted.

## Conclusion

The deployment of MST personnel as field supervisors was followed by apparent improvement in the quality of polio vaccination campaigns as measured by vaccination coverage in post campaign LQAS surveys. This effort, in collaboration with many other efforts and interventions, like the application of accountability framework to health workers by the government and other PEI partners, was followed by substantial reduction in the number of wild polio virus (WPV) cases in the country from one hundred and twenty-two cases in 2012 to six cases in 2014, then to four cases in 2016 and zero cases afterwards to date. Given the success of the MST in improving immunization coverage, the training and deployment of MST personnel will continue until global eradication of WPV is achieved. Accordingly, similar approach could be expanded to the control other VPDs.

### What is known about this topic

- The Nigerian polio eradication initiative effort is coordinated by the national polio emergency operation center (NEOC);
- NEOC was established in 2012 and coordinates the activities of all GPEI partners to fight the scourge of the poliovirus;
- National Stop Transmission of Polio (NSTOP) in collaboration with NEOC and other partners deployed MSTs for every round of supplemental immunization activities (SIAs) to ensure quality supervision.

### What this study adds

- The concept and strategy of management support team (MST) deployment during vaccination campaigns to supervise vaccination teams and ensure quality of campaign can be adopted by other polio endemic countries and other VPDs targeted for control and elimination or eradication.
